# Molekularpathologie des nichtkleinzelligen Lungenkarzinoms: aktuelle und kommende Biomarker

**DOI:** 10.1007/s00292-025-01433-x

**Published:** 2025-04-03

**Authors:** Helen Pasternack, Jutta Kirfel

**Affiliations:** Institut für Pathologie, Universitätsklinikum Schleswig-Holstein, Campus Lübeck, Ratzeburger Allee 160, 23562 Lübeck, Deutschland

**Keywords:** Präzisionsmedizin, NSCLC, Prädiktive Testung, Molekulare zielgerichtete Therapie, Next Generation Sequencing, Precision medicine, NSCLC, Predictive testing, Molecular targeted therapy, Next generation sequencing

## Abstract

In der Klassifikation des Lungenkarzinoms gilt weiterhin die grundsätzliche Einteilung nach kleinzelligen und nichtkleinzelligen Karzinomen (NSCLC). Trotz gleicher histologischer Subtypisierung ist bekannt, dass es definierte genetische Veränderungen in den Tumorzellen gibt. Diese bestimmen im Sinne von „Treibern“ das Tumorwachstum maßgeblich, sodass ihre Blockade den klinischen Verlauf erheblich beeinflussen kann. So wurde die Therapie des NSCLC in den letzten 10 Jahren zunehmend durch die Etablierung tumorspezifisch zielgerichteter Medikamente und immunmodulatorischer Ansätze ergänzt und hat dadurch rasant an Komplexität gewonnen. Diese Entwicklung führte zu einem immer differenzierteren und zunehmend individualisierten Vorgehen in der Behandlung. Der Pathologie und insbesondere der molekularpathologischen Diagnostik kommt dabei eine zentrale Rolle zu, da hier eine zunehmende Anzahl von Biomarkern untersucht werden muss.

## Abstract (en)

In the classification of lung cancer, the basic division into small cell and non-small cell carcinomas continues to apply. Despite the same histological subtyping, it is known that there are defined genetic changes in the tumor cells that significantly determine tumor growth as “drivers”, so that their blockade can significantly influence the clinical course. Thus, in the last 10 years, the treatment of lung cancer has been increasingly supplemented by the establishment of tumor-specific targeted drugs and immunomodulatory approaches. This development has led to increasingly differentiated and individualized approaches to treatment. Pathology and especially molecular pathological diagnostics play a central role here, as an increasing number of biomarkers must be examined.

## Lernziele

Nach Lektüre dieses Beitrags …

- können Sie die obligat zu testenden Biomarker für die zugelassenen zielgerichteten Therapien im nichtkleinzelligen Lungenkarzinom („non-small cell lung cancer“, NSCLC) benennen;
- kennen Sie die grundlegenden molekularpathologischen Untersuchungsmethoden dieser Biomarkertestung;
- können Sie weitere potenzielle Biomarker einordnen;
- ziehen Sie Schlüsse aus den Ergebnissen der molekularpathologischen Testungen;
- können Sie die klinisch tätigen Kollegen auf Basis der molekularpathologischen Befunde bezüglich der weiteren möglichen Therapieoptionen beraten.

## Hintergrund

Lungenkarzinome gehören in Deutschland zu den häufigsten malignen Erkrankungen und sind die häufigste krebsbedingte Todesursache. Sie werden histologisch in kleinzellige und nichtkleinzellige Karzinome unterschieden. Nach der aktuellen Klassifikation der Weltgesundheitsorganisation (WHO) zählen zu den NSCLC („non-small cell lung cancer“) folgende **histologische Typen**

histologische Typen:

Adenokarzinome (etwa 50 %), Plattenepithelkarzinome (etwa 25 %), großzellige Karzinome (etwa 10 %) und die Gruppe der anderen und unspezifizierten (O & U) Karzinome [1]. Epidemiologische Studien lassen Rückschlüsse auf eine Steigerung des auf das NSCLC bezogenen Überlebens zu, die nicht allein durch eine moderate Reduktion der Inzidenz, sondern auch durch die Erfolge der **„targeted therapies“**

„targeted therapies“

zu erklären sind [2]. Gleiches gilt nicht für das kleinzellige Lungenkarzinom (SCLC), für das bis dato keine zielgerichteten Therapien zur Verfügung stehen. Die wirksamste Behandlungsform beim SCLC sind Chemotherapie und Immuntherapie. Jedes NSCLC verlangt hingegen ab dem frühestmöglichen Zeitpunkt in der Diagnosefindung nach einer **molekularen Analyse**

molekularen Analyse

auf therapierelevante Alterationsmuster.

Zur Festlegung der personalisierten Therapie gehört neben der histologischen Typisierung des NSCLC und dem Ausbreitungsgrad eine molekulare Charakterisierung, die Eingang in die **Leitlinienempfehlungen**

Leitlinienempfehlungen

gefunden hat [3, 4, 5]. Sie erfolgt heutzutage standardmäßig weitgehend unabhängig vom jeweiligen histologischen Subtyp („histologieagnostisch“). Durch das **Next Generation Sequencing**

Next Generation Sequencing

(NGS) als schnelles und umfassendes Sequenzierungsverfahren auf DNA- und RNA-Ebene gelangen zunehmend mehr Alterationen in den Fokus, wenngleich noch nicht für alle entsprechende Pharmaka zur Verfügung stehen.

Die **prädiktiven Biomarker**

prädiktiven Biomarker

für das NSCLC lassen sich grob in 3 Kategorien einteilen, je nachdem, inwieweit ihr Nachweis mit einem Ansprechen auf ein Medikament verbunden ist:

1. routinemäßig untersuchte (obligate) Biomarker mit Alterationen von unmittelbarer klinischer Relevanz;
2. neue Biomarker, die noch nicht in der klinischen Routine sind, aber empfohlen werden und
3. Biomarker, die derzeit eher noch explorativen Charakter haben.

Zusätzlich unterscheidet man die Biomarker, je nachdem ob sie prädiktiv für die zielgerichtete Therapie oder das Ansprechen auf eine **Immuncheckpointblockade**

Immuncheckpointblockade

(häufig auch nur als Immuntherapie bezeichnet) sind [6, 7]. Einen Überblick über die wichtigsten Biomarker gibt Tab. 1.

**Tab. 1:** Biomarker für die indikationsbezogene prädiktive Diagnostik^a^

Anwendung	Aktuelle Biomarker		Neue empfohlene Biomarker	
Prädiktiv für zielgerichtete Therapie	ALK^b,1,2,3^	Fusion/Überexpression Resistenzmutation	BRAF	Non-V600-Mutation
	BRAF^1,2,3^	V600-Mutation	BRCA 1/2	Funktionsverlustmutation
	EGFR^b,1,2,3^	Aktivierende Mutation Resistenzmutation	ERBB2^1,3^	Amplifikation/Überexpression
	ERBB2^1,2,3^	Aktivierende Mutation	FGFR1–4	Amplifikation/Überexpression Fusion Aktivierende Mutation
	KRAS^1,2,3^	G12C-Mutation	MAP2K1	Aktivierende Mutation
	MET^1,2,3^	Exon-14-Skipping-Mutation	MET^1,2^	Amplifikation/Überexpression, Fusion
	NTRK1/2/3^1,2,3^	Fusion/Überexpression	NRG1^1,2,3^	Fusion
	RET^1,2,3^	Fusion Resistenzmutation	PIK3CA	Aktivierende Mutation
	ROS1^1,2,3^	Fusion/Überexpression Resistenzmutation	TP53	Funktionsverlustmutation
Prädiktiv für Immuntherapie	PD-L1^b,1,2,3^	Proteinexpression (immunhistochemischer Marker, keine molekularpathologische Testung)	KEAP1	Funktionsverlustmutation
			NFE2L2	Aktivierende Mutation
			NOTCH	Aktivierende Mutation
			SMARCA4	Funktionsverlustmutation
			STK11	Funktionsverlustmutation
			TMB	Tumormutationslast

^a^Aktuelle Biomarker mit zugelassener Therapieoption sowie neue empfohlene Biomarker mit derzeit noch nicht zugelassener Therapieoption

^b^Testung bereits im operablen Stadium

^1,2,3^Explizite Empfehlungen durch: ^1^S3-Leitlinie [8], ^2^European Society for Medical Oncology (ESMO; [9]) oder ^3^National Comprehensive Center Network (NCCN; [10])

## Alterationen von unmittelbarer klinischer Relevanz

Die Diagnostik therapierelevanter Alterationen und Biomarker soll bei allen Patient*innen in den operablen Stadien für diese Marker erfolgen:

- PD-L1-Protein-Expression;
- *ALK-*Fusion/-Überexpression;
- *EGFR*-Mutationen in den Exons 18–21.

Hintergrund dieser Empfehlung ist zum einen, dass der Zulassungsstatus der adjuvanten Immuntherapie auf dem Nachweis der PD-L1-Protein-Expression beruht und Patient*innen mit *EGFR*- und *ALK*-Alterationen ausschließt. Zum anderen kann bei Nachweis entsprechender Alterationen in *EGFR* oder *ALK* auch in frühen Stadien eine zielgerichtete Therapie indiziert sein. Zusätzlich wird eine umfassende molekulare Testung wie im fortgeschrittenen Stadium diskutiert, da auch Patient*innen mit weiteren Alterationen, z. B. in *ROS1* oder *RET*, von einer perioperativen Immun‑, Immunchemo- oder zielgerichteten Therapie profitieren könnten [3].

**Abb. 1:**
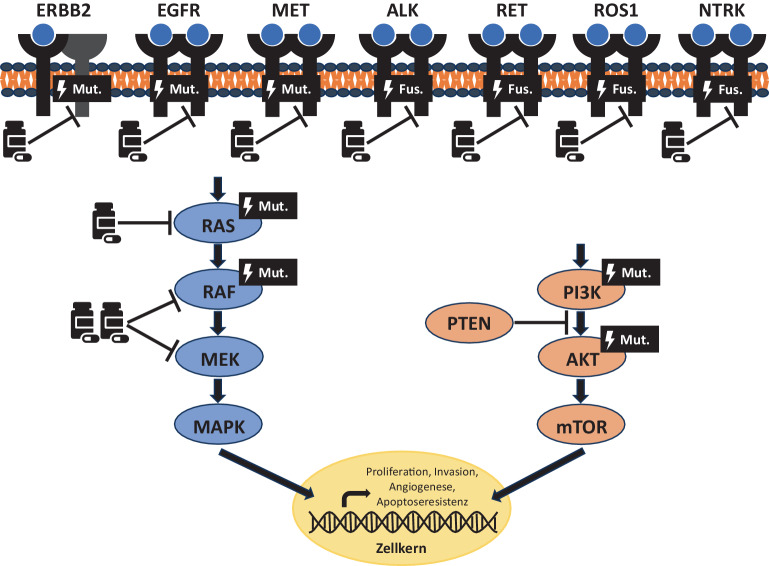
Zugelassene zielgerichtete Therapieoptionen und wichtige beteiligte Signalwege im nichtkleinzelligen Lungenkarzinom (NSCLC). *Mut.* Mutation, *Fus.* Fusion

Die Diagnostik therapierelevanter Alterationen soll bei allen Patient*innen im fortgeschrittenen Stadium vor Beginn einer medikamentösen Erstlinientherapie erfolgen. Sie soll den immunhistochemisch bestimmten PD-L1-Status sowie folgende Aberrationen erfassen: *ALK*-Translokationen, *BRAF*-V600E-Mutation, *EGFR*-Mutationen in den Exons 18–21 , *ERBB2*-Mutationen, *KRAS*-G12C-Mutation, *MET-*Exon-14-Skipping-Mutationen, *NTRK-*Translokationen, *RET-*Translokationen und *ROS1-*Translokationen [3]. Die Abb. 1 gibt einen Überblick der genannten Biomarker im zellulären Kontext.

**Translokationen**

Translokationen

oder eine Inversion in *ALK* („anaplastic large cell lymphoma receptor tyrosine kinase“) kommen in etwa 4 % der NSCLC-Fälle in Europa und Amerika vor [11]. Sie führen zu dessen **dauerhafter Aktivierung**

dauerhafter Aktivierung

und wirken über mehrere nachgeschaltete Signalwege, wie RAS/RAF/MEK und die PI3K/AKT-Signalkaskade, onkogen. Häufigster **Translokationspartner**

Translokationspartner

ist das *EML4*-Gen („echinoderm microtubule-associated protein-like 4“), das durch die Fusion zu einer Überexpression von ALK führt. Neben *EML4* gibt es noch weitere Fusionspartner für *ALK*; abhängig vom jeweiligen Fusionspartner wird ein chimäres Protein mit unterschiedlicher Biologie gebildet und in Folge zeigen die betroffenen Patient*innen ein variables Ansprechen auf **Tyrosinkinaseinhibitoren**

Tyrosinkinaseinhibitoren

(TKI; [12]). Für die Therapie von Patient*innen mit *ALK*-Translokationen stehen derzeit mehrere zugelassene TKI zur Verfügung. Allerdings können unter Therapie **Resistenzmutationen**

Resistenzmutationen

in der Kinasedomäne von *ALK* oder anderen Genen (z. B. *EGFR*) auftreten. Bei Progress unter einem ALK-Inhibitor sollte der Resistenzmechanismus durch eine Geweberebiopsie untersucht werden.

Das Protoonkogen *BRAF* kodiert für die Serin-Threonin-Proteinkinase B‑RAF. Diese ist Bestandteil des MAP-Kinase/ERK-Signalwegs und an der intrazellulären Signaltransduktion von **Wachstumssignalen**

Wachstumssignalen

beteiligt. Eine p.V600E-Mutation tritt in etwa 2 % der NSCLC auf und führt zu einer Aktivierung des Proteins, des Signalwegs und somit zum Tumorwachstum [11]. Bei Nachweis einer *BRAF*-V600-Mutation können die Patient*innen von einer zielgerichteten Therapie mit einer Kombination aus einem BRAF- und einem MEK-Inhibitor profitieren.

Der Epidermal-growth-factor(EGF)-Rezeptor ist eine Rezeptortyrosinkinase und dimerisiert nach Bindung des EGF. Nachfolgend führt dies zur **Autophoshorylierung**

Autophoshorylierung

der intrazellulären Tyrosinkinasedomäne und einer Aktivierung sowohl der RAS-RAF-MEK- als auch der PI3K-AKT-Signalkaskade. Mutationen im *EGFR*-Gen können zu einer konstanten Signalwegaktivierung führen. Klinisch haben sich bei NSCLC-Patient*innen mit dem Nachweis einer aktivierenden *EGFR*-Mutation mehrere EGFR-TKI bewährt. Die häufigsten Mutationen im NSCLC stellen dabei mit etwa 15 % Deletionen in Exon 19 sowie die Punktmutation p.L858R in Exon 21 des *EGFR*-Gens dar [11]. Bei Patient*innen mit anderen *EGFR*-Aberrationen in den Exons 18–21 („uncommon mutations“) ist das Ansprechen gegenüber EGFR-TKI sehr heterogen und abhängig von der jeweils resultierenden EGFR-Proteinstruktur sowie der Wahl des einzelnen TKI [13]. Bei Progress unter TKI und Verdacht auf Resistenz sollte der Resistenzmechanismus durch eine Geweberebiopsie bzw. durch eine **Liquid Biopsy**

Liquid Biopsy

umfassend untersucht werden, um eine andere, zielgerichtet behandelbare Alteration zu identifizieren. Zu möglichen Resistenzmechanismen zählen dabei u. a. eine *MET*- oder *ERBB2*-Amplifikation sowie eine unabhängige Aktivierung des MAPK- oder PI3K-Signalwegs [14].

Das Gen* ERBB2 (HER2)* ist ein Mitglied der ERBB-Familie der Tyrosinkinaserezeptoren. Da das ERBB2-Protein keine eigene Ligandenbindungsdomäne besitzt, kann es keine Wachstumsfaktoren binden, sondern bildet **Heterodimere**

Heterodimere

mit anderen ligandenbindenden Mitgliedern der ERBB-Rezeptorfamilie und verstärkt so die kinasevermittelte Aktivierung von Signaltransduktionswegen. Alterationen in *ERBB2/HER2* führen zur Aktivierung einer Vielzahl von Signalwegen, darunter MAPK und PI3K/AKT. Generell können *ERBB2*-Abberationen beim NSCLC als **Onkogene**

Onkogene

in der Primärsituation, aber auch bei erworbener Resistenz nach einer gezielten Therapie identifiziert werden. Etwa 2 % der NSCLC-Patient*innen weisen eine aktivierende *ERBB2*-Mutation auf [11]. Sie können bei Progression nach platinbasierter Chemotherapie (mit oder ohne Immuntherapie) mit dem **Antikörper-Wirkstoff-Konjugat**

Antikörper-Wirkstoff-Konjugat

Trastuzumab-Deruxtecan behandelt werden. *ERBB2*-Amplifikationen sind einer der häufigsten erworbenen Resistenzmechanismen unter EGFR-TKI-Therapie, als Zielstruktur für eine zugelassene zielgerichtete Therapie im NSCLC spielen sie hingegen aktuell keine Rolle.

Das Protoonkogen *KRAS* („kirsten rat sarcoma viral oncogene homolog“) kodiert für die GTPase KRAS. Diese ist Bestandteil des MAP-Kinase/ERK-Signalwegs und an der intrazellulären Signaltransduktion von Wachstumssignalen beteiligt. Eine entsprechende Mutation, wie die im NSCLC mit etwa 15 % häufigste p.G12C-Mutation, führt zu einem dauerhaften Verbleib des G‑Proteins im aktiven GTP-gebundenen Zustand [11]. Der so konstitutiv aktive Signalweg fördert in der Tumorzelle Proliferation, Angiogenese und Metastasierung. Bei Nachweis einer p.G12C-Mutation im *KRAS*-Gen können die Patient*innen von einer zielgerichteten Therapie mit einem KRAS-G12C-Inhibitor profitieren.

Das *MET*-Gen kodiert für die transmembranäre Rezeptortyrosinkinase MET („mesenchymal–epithelial transition factor“), die durch ihren Liganden HGF („hepatocyte growth factor“) aktiviert wird und unter anderem über die MAP-Kinase/ERK- und PI3-Kinase-Signalwege an Wachstumssignalen beteiligt ist. Verschiedene Mechanismen können im NSCLC zu einer konstitutiven MET-Aktivierung führen. Dabei weisen etwa 2 % der Patient*innen eine sog. *MET*-Exon-14-Skipping-Mutation auf, bei der verschiedene Mutationen in den Spleißregionen zu einem Verlust von Exon 14 im MET-Protein führen [11]. Dieses verkürzte Protein weist eine erhöhte Stabilität auf und bewirkt so eine Aktivierung der nachgeschalteten Signalübertragung. Bei Nachweis von Exon-14-Skipping in *MET* können die Patient*innen von einer zielgerichteten Therapie mit TKI profitieren. Eine *MET*-Genamplifikation hingegen spielt derzeit als Zielstruktur für eine zugelassene zielgerichtete Therapie im NSCLC keine Rolle, stellt aber einen der häufigsten erworbenen Resistenzmechanismen unter EGFR-TKI-Therapie dar.

Die Tropomyosinrezeptorkinasen A, B und C (TRKA, TRKB und TRKC), kodiert durch die Gene *NTRK1, NTRK2* und *NTRK3,* sind Rezeptortyrosinkinasen. Die *NTRK*-Genfusionen entstehen durch intrachromosomale oder interchromosomale Genumlagerungen zwischen der 3’-Region des *NTRK*-Gens und der 5’-Region des Fusionspartnergens. Mit einer Frequenz von etwa 0,3 % im NSCLC sind *NTRK*-Fusionen selten, aber betroffene Patient*innen können von einer zielgerichteten Therapie mit einem selektiven TKI profitieren [11].

Das *RET*-Gen kodiert für eine transmembranäre Rezeptortyrosinkinase, die unter anderem über die MAP-Kinase/ERK- und PI3-Kinase-Signalwege an der Signaltransduktion von Wachstumssignalen beteiligt ist. Im NSCLC kommen in etwa 1 % der Fälle intra- und interchromosomale Genfusionen von *RET* mit zahlreichen unterschiedlichen Partnern vor [11]. Die verschiedenen *RET*-Translokationen führen zu einer Fusion der 3’-Kinasedomäne von *RET* mit heterologen 5’-Gensequenzen und so zu einer Aktivierung intrazellulärer Signalwege, die beispielsweise Proliferation, Angiogenese und Metastasierung fördern. Bei Nachweis einer *RET*-Genumlagerung können die Patient*innen von einer zielgerichteten Therapie mit TKI profitieren. Mögliche Resistenzmechanismen unter RET-TKI-Therapie sind dabei beispielsweise Mutationen im *RET*-Gen (Codon G810) oder Amplifikationen des *KRAS*- oder *MET*-Gens.

Die Rezeptortyrosinkinase *ROS1* („ROS proto-oncogene 1“) ist ein Mitglied der Insulinrezeptorfamilie und fungiert als Wachstums- oder **Differenzierungsfaktorrezeptor**

Differenzierungsfaktorrezeptor.

In etwa 2 % der NSCLC-Fälle können Genfusionen unter Beteiligung von *ROS1* nachgewiesen werden, die eine aberrante ROS1-Kinaseaktivität bewirken und so zur Aktivierung zentraler nachgeschalteter Signalwege, wie RAS-MAPK/ERK- und AKT/mTOR, führen [11]. Bei Patient*innen mit den seltenen aktivierenden *ROS1*-Translokationen sind 2 TKI in der Erstlinientherapie zugelassen. Bei Progress unter TKI und Verdacht auf Resistenz sollte der Resistenzmechanismus durch eine Geweberebiopsie umfassend untersucht werden, insbesondere mit der Frage nach einer *ROS1*-G2032R-Resistenzmutation oder einer anderen, zielgerichtet behandelbaren Alteration.

### Merke

Die Diagnostik therapierelevanter Biomarker soll bei allen Patient*innen in den operablen Stadien umfassen: PD-L1-Protein-Expression, ***ALK-***Fusion/-Überexpression, ***EGFR***-Mutationen in den Exons 18–21 . Bei allen Patient*innen im fortgeschrittenen Stadium soll die prädiktive Diagnostik erfolgen für: PD-L1-Protein-Expression, ***ALK***-Translokationen, ***BRAF***-V600E-Mutation, ***EGFR***-Mutationen in den Exons 18–21 , ***ERBB2***-Mutationen, ***KRAS***-G12C-Mutation, ***MET-***Exon-14-Skipping-Mutationen, ***NTRK-***Translokationen, ***RET-***Translokationen und ***ROS1-***Translokationen.

## Neue empfohlene Biomarker

Einige Biomarker werden in der täglichen Routine noch nicht systematisch ausgewertet, werden aber empfohlen (Tab. 1). Diese Biomarker werden schon in klinischen Studien untersucht oder ermöglichen ggf. eine **Off-Label-Therapie-Option**

Off-Label-Therapie-Option.

Es handelt sich um *BRAF*-nicht-V600-Mutationen, Funktionsverlustmutationen in *BRCA 1/2*, Amplifikationen in *ERBB2 (HER2)*, aktivierende *MAP2K1-*Mutationen, Amplifikationen und Translokationen in *MET*, Fusionen in *NRG1*, aktivierende Mutationen in *PIK3CA* (PI3K-katalytische Untereinheit Alpha) sowie *TP53*-Funktionsverlustmutationen [3, 15, 16, 17, 18, 19, 20, 21]. Darüber hinaus wurden insbesondere im Plattenepithelkarzinom der Lunge Fusionen, Mutationen und Amplifikationen in den Genen *FGFR1–4* identifiziert [22].

Andere Biomarker werden im Zusammenhang mit der Vorhersage der primären Resistenz gegen **Immuncheckpointinhibitoren**

Immuncheckpointinhibitoren

(ICI) bewertet. Dabei sind Funktionsverlustmutationen in *KEAP1*, aktivierende *NFE2L2*-Mutationen, aktivierende Mutationen in *NOTCH* und Funktionsverlustmutationen in *SMARCA4* sowie *STK11* zu nennen [23, 24, 25, 26, 27, 28, 29]. Die Testung der **Tumormutationslast**

Tumormutationslast

(TMB) ist ein weiterer potenzieller prädiktiver Biomarker, um NSCLC-Patienten zu identifizieren, die von einer ICI-Therapie profitieren [30]. Die TMB ist definiert als die Anzahl der Mutationen pro Megabase analysierte DNA.

Die genannten *BRCA-1*/*2*-Mutationen und Mutationen in weiteren DNA-Reparaturgenen sind Ursache für eine **homologe Rekombinationsdefizienz**

homologe Rekombinationsdefizienz

(HRD). Die dysfunktionale DNA-Reparatur führt zu genomischer Instabilität, die das Tumorwachstum beschleunigt. Eine HRD wird indirekt nachgewiesen: zum einen durch Mutationsanalysen relevanter **Reparaturgene**

Reparaturgene,

zum anderen über die Bestimmung des aus 3 Faktoren zusammengesetzten HRD-Scores („loss of heterozygosity“ [LOH], „large scale state transitions“ [LST] und „telomeric allelic imbalance“ [TAI]). Ob sich der HRD-Score beim NSCLC als prädiktiver Biomarker für das Ansprechen auf Poly-ADP-Ribose-Polymerase (PARP)-Inhibitoren als Mono- oder Kombinationstherapie eignet, müssen weitere prospektive klinische Studien zeigen [31, 32].

### Merke

Zusätzlich zu den obligat zu testenden Biomarkern werden weitere Marker empfohlen, die entweder prädiktiv für eine zielgerichtete Therapie oder eine Immuncheckpointblockade sind.

## Explorative Biomarker

In präklinischen Studien werden zunehmend weitere potenzielle Biomarker untersucht [6]. Zu diesen derzeit noch explorativen Markern gehören andere aktivierende Mutationen in den genannten zentralen intrazellulären MAPK- und PI3K-Signalwegen. Hier sind z. B. Mutationen im *AKT1*-Gen („AKT serine/threonine kinase 1“ oder häufig auch Proteinkinase B), in *HRAS* („HRAS proto-oncogen, GTPase“) oder *NRAS* („NRAS proto-oncogen, GTPase“) zu nennen [33, 34]. Funktionsverlustmutationen hingegen finden sich im Tumorsuppressorgen *PTEN* („phosphatase and tensin homolog“), das für eine Phosphatase kodiert, die ebenfalls an der PI3K/AKT/mTOR-Signalkaskade beteiligt ist [34].

Auch in weiteren intrazellulären Signalwegen wurden aktivierende Genmutationen im NSCLC identifiziert. Dazu zählen *CTNNB1* („catenin beta 1“), das für das **β‑Catenin-Protein**

(‑Catenin-Protein

mit entscheidender Rolle im Wnt-Signalweg kodiert, sowie die intrazellulären Tyrosinkinasen *JAK 2/3* (Januskinase 2 und 3). Auch in den Isozitratdehydrogenasen 1 und 2, kodiert durch die *IDH1*- und *IDH2*-Gene, können aktivierende Mutationen detektiert werden. Einen weiteren potenziellen Biomarker im NSCLC stellt das bekannte Tumorsuppressorgen *RB1* („RB transcriptional corepressor 1“ oder auch „retinoblastoma 1“) dar, in dem Funktionsverlustmutationen nachgewiesen werden können [35].

Als zusätzlicher möglicher prädiktive Biomarker für die Immuntherapie werden *CUL3*(Cullin-3)-Funktionsverlustmutationen genannt [29].

### Merke

In Zukunft werden weitere Biomarker Einzug in die molekularpathologische Testung finden, die derzeit Gegenstand klinischer und präklinischer Forschung sind.

## Methoden der Biomarkertestung

Die molekularpathologische Analyse hinsichtlich aller therapeutisch relevanten Genveränderungen soll vor Erstlinientherapie eingeleitet werden. Hierzu kann grundsätzlich jede Art von histologischem oder zytologischem Material verwendet werden. Wenn eine Tumorprobe nicht in ausreichender Menge vorliegt, kann zirkulierende **zellfreie DNA**

zellfreie DNA

(ccfDNA) für die Mutationstestung verwendet werden, die aus einer Blutprobe gewonnen wird (Liquid Biopsy).

Der Nachweis von Mutationen auf DNA-Ebene kann durch verschiedene Methoden als Einzelgen- oder im Rahmen einer **Paneltestung**

Paneltestung

erfolgen. Da das Untersuchungsmaterial, insbesondere bei kleinen Biopsien oder Zytologien, limitiert ist und um eine zeitnahe Analyse aller relevanten Marker zu gewährleisten, empfiehlt sich eine gleichzeitige Mutationsanalyse mehrerer Gene als Paneltestung mittels Parallelsequenzierung (auch NGS). Dabei wird üblicherweise eine **minimale Mutationsfrequenz**

minimale Mutationsfrequenz

von etwa 5 % detektiert und berichtet, so dass die zu untersuchende Tumorprobe einen **Tumorzellanteil**

Tumorzellanteil

(Anteil der Tumorzellen an allen kernhaltigen Zellen im Präparat) von mindestens etwa 10 % haben sollte. Ein geeignetes Tumorareal mit möglichst hohem Tumorzellanteil sollte von erfahrenen Patholog*innen ausgewählt und mittels Dissektion gezielt für die DNA-Extraktion eingesetzt werden.

Der Nachweis einer Expression/Überexpression erfolgt mittels **Immunhistochemie**

Immunhistochemie.

Die Exon 14-Skipping-Mutationen in *MET* können durch verschiedene Methoden als Einzelgen- oder im Rahmen einer Paneltestung detektiert werden. Eine separate Mutationsanalyse nur für *MET*-Exon-14-Skipping kann beispielsweise mittels quantitativer Polymerasekettenreaktion mittels reverser Transkriptase (qRT-PCR) durchgeführt werden. Es empfiehlt sich aber ein *MET*-Exon 14-Skipping-Nachweis gleichzeitig mit der Analyse weiterer Gene mittels NGS als Paneltestung auf DNA- und RNA-Ebene.

Die Detektion einer Genumlagerung in *ALK, RET, ROS1 *oder einem der *NTRK*-Gene kann durch verschiedene Methoden, wie **Fluoreszenz-in-situ-Hybridisierung**

Fluoreszenz-in-situ-Hybridisierung

(FISH), NGS und qRT-PCR, gelingen. Auch hier empfiehlt sich aber ein paralleler Nachweis zusammen mit weiteren Genen als NGS-Paneltestung auf DNA- und/oder RNA-Ebene. Für *ALK* ist mit geeigneten Antikörpern auch ein indirekter immunhistochemischer Nachweis möglich.

Für den Nachweis einer der genannten Genumlagerungen bzw. von *MET*-Exon-14-Skipping weist eine Testung auf RNA-Ebene gegenüber einer auf DNA-Ebene eine höhere Sensitivität auf.

Amplifikationen, wie z. B. eine *ERBB2*-Amplifikation, können mittels FISH oder einer DNA-basierten Parallelsequenzierung nachgewiesen werden. Als indirekter Nachweis kann zudem die gesteigerte Proteinexpression immunhistochemisch detektiert werden.

Für eine ccfDNA-Analyse anhand einer Liquid Biopsy ist eine besonders hohe Sensitivität notwendig, um die zum Teil nur sehr geringen Mengen an zirkulierender Tumor-DNA (ctDNA) nachzuweisen. Hier kommt eine Untersuchung mittels sensitiver Parallelsequenzierung oder z. B. „Digitaler-droplet“(dd)-PCR infrage. In einer Metaanalyse (17 Studien) zur Detektion von *EGFR-*Mutationen beim NSCLC mithilfe von Liquid Biopsy wurde gezeigt, dass die Sensitivität und Spezifität am höchsten bei fortgeschrittenen Krankheitsstadien waren und sich NGS- und PCR-basierte Techniken (ddPCR, Peptide-nucleic-acid[PNA]-locked-nucleic-acid[LNA]-PCR), Amplification-refractory-mutation-system[ARMS]-PCR) als gleichermaßen praktikabel erwiesen. Die Sensitivität in der Metaanalyse lag bei 59 %, die Spezifität bei 96 % [36]. Für Liquid-Biopsy-Analysen wird auch durch die entsprechend anzusetzenden Abrechnungsziffern gemäß einheitlichem Bewertungsmaßstab (EBM) eine besonders niedrige Nachweisgrenze („limit of detection“, LOD) mit einer 0,1 oder 0,5 %igen Mutationsfrequenz gefordert.

### Cave

Die eingesetzten molekularpathologischen Methoden müssen für die jeweiligen Biomarker im Hinblick auf die nachzuweisenden Alterationen und erforderliche Sensitivität sorgfältig ausgewählt werden.

## Fazit für die Praxis

- Die Etablierung zielgerichteter Therapieoptionen im nichtkleinzelligen Lungenkarzinom (NSCLC) war ein Paradigmenwechsel.
- Heute darf nicht nur keinem Patienten eine molekularpathologische Testung auf klinisch relevante Genalterationen im Tumor vorenthalten werden; das Diagnostik- und Behandlerteam muss sich auch mit den ständigen Neuerungen auf diesem Gebiet auseinandersetzen.
- Gemäß aktueller Leitlinien und der Verfügbarkeit zugelassener zielgerichteter Therapiekonzepte sollen als prädiktive Biomarker im operablen Stadium eines NSCLC mindestens getestet werden: PD-L1-Proteinexpression, ***ALK***-Fusion/-Überexpression und ***EGFR***-Mutation. Im fortgeschrittenen Stadium sollen zusätzlich untersucht werden: ***BRAF-***V600-Mutation, ***ERBB2***-Mutation, ***KRAS***-G12C-Mutation, ***MET***-Exon 14-Skipping-Mutation, ***NTRK1/2/3***-Fusion/-Überexpression, ***RET***-Fusion und ***ROS1***-Fusion/-Überexpression.
- Die Molekularpathologie nimmt beim NSCLC eine zentrale Rolle ein, da hier entscheidende Biomarker für die Indikationsstellung zielgerichteter Therapien getestet werden müssen.

## CME-Fragebogen

Sie haben ein primäres Adenokarzinom der Lunge im Stadium IIB gemäß Union Internationale Contre le Cancer (UICC) diagnostiziert. Welche prädiktiven Marker sollen mindestens untersucht werden?

PD-L1, *ALK* und NTRK

PD-L1, *EGFR* und *BRAF*

*EGFR, ALK* und *KRAS*

PD-L1, *EGFR* und *ALK*

*EGFR, ERBB2 *und NTRK

Bei einem nichtkleinzelligen Lungenkarzinom (NSCLC) im fortgeschrittenen Stadium IV gemäß Union Internationale Contre le Cancer (UICC) liegt der PD-L1-Status bereits vor, die Untersuchung welcher weiteren prädiktiven Marker soll mindestens ergänzt werden?

*EGFR, KRAS, ALK, RET *und *ROS1*

*BRAF, EGFR, ERBB2, KRAS, MET-*Exon-14-Skipping, NTRK, *ALK, RET *und *ROS1*

*BRAF, EGFR, MET-*Exon-14-Skipping und *ALK*

*BRCA 1/2, EGFR, ERBB2, TP53, MET-*Exon-14-Skipping, NTRK, *ALK *und *ROS1*

*EGFR, ERBB2, KRAS, MET-*Exon-14-Skipping, *PIK3CA, TP53, ALK, NRG1* und *ROS1*

Eine klinische Kollegin bittet Sie vor Einleitung der Erstlinientherapie um die Testung prädiktiver Marker in einem pulmonalen Adenokarzinom. Welche Methode ist für die Testung von *EGFR* zur Indikationsstellung einer zugelassenen zielgerichteten Therapieoption am ehesten geeignet?

Fluoreszenz-in-situ-Hybridisierung

Immunhistochemie

Sanger-Sequenzierung von Exon 20 und 21

Quantitative Polymerasekettenreaktion mittels reverser Transkriptase (qRT-PCR)

DNA-basiertes Next-Generation-Sequencing(NGS)-Panel

Mit welchen Methoden können beim nichtkleinzelligen Lungenkarzinom (NSCLC) in einer „Liquid Biopsy“ therapierelevante Mutationen auf DNA-Ebene am ehesten detektiert werden?

„Digitale-droplet“-PCR (ddPCR) oder Next Generation Sequencing (NGS)

Sanger-Sequenzierung oder Next Generation Sequencing (NGS)

Quantitative Polymerasekettenreaktion mittels reverser Transkriptase (qRT-PCR) oder Fluoreszenz-in-situ-Hybridisierung

„Digitale-droplet“-PCR (ddPCR) oder Immunhistochemie

Next Generation Sequencing (NGS) oder Fluoreszenz-in-situ-Hybridisierung

In Fall eines nichtkleinzelligen Lungenkarzinoms (NSCLC) wurde im Rahmen der prädiktiven Analytik ein erweitertes Next-Generation-Sequencing(NGS)-Panel untersucht. Dabei wurde eine Funktionsverlustmutation im *STK11*-Gen nachgewiesen. Welche Information können Sie darauf basierend an die klinischen Kolleg*innen weitergeben?

Die detektierte *STK11*-Mutation führt zu einer primären Resistenz gegenüber EGFR-Tyrosinkinaseinhibitoren.

Es besteht eine zugelassene Therapieoption mit einem STK11-Tyrosinkinaseinhibitor.

Die detektierte *STK11*-Mutation kann einen Einfluss auf das Ansprechen gegenüber einer Immuncheckpointinhibitortherapie haben.

Eine zielgerichtete Therapieoption mit einem gegen STK11 gerichteten Antikörper im Rahmen von klinischen Studien ist zu prüfen.

Die detektierte *STK11*-Mutation hat nach derzeitigem Kenntnisstand keinerlei Einfluss auf eine medikamentöse Systemtherapie.

Welche therapeutische Konsequenz kann bei Vorliegen einer *KRAS-*p.G12C-Mutation in einem nichtkleinzelligen Lungenkarzinom (NSCLC) abgeleitet werden?

Es besteht eine zugelassene Therapieoption mit einem spezifischen KRAS-G12C-Inhibitor.

Die detektierte *KRAS*-Mutation hat nach derzeitigem Kenntnisstand keinerlei Einfluss auf eine medikamentöse Systemtherapie.

Eine zielgerichtete Therapieoption mit einem KRAS-Inhibitor besteht nur im Rahmen von klinischen Studien.

Bei Vorliegen einer *KRAS*-Mutation ist eine Immuncheckpointinhibitortherapie unabhängig vom PD-L1-Status indiziert.

Die p.G12C-Mutation führt zu einer Inaktivierung des KRAS-Proteins, sodass eine intensivierte Chemo- und Strahlentherapie notwendig ist.

Für welche Veränderung im *MET*-Gen besteht aktuell eine zugelassene Therapieoption beim nichtkleinzelligen Lungenkarzinom (NSCLC) ?

*MET*-Amplifikation

*MET*-Translokation

*MET*-Funktionsverlustmutation

*MET*-Exon-14-Skipping

*MET*-Überexpression

Bei welchem der folgenden Biomarker kann bei Vorliegen einer Funktionsverlustmutation am ehesten ein Einfluss auf das Ansprechen gegenüber einer Immuncheckpointinhibitortherapie erwartet werden?

BRAF

ERBB2

BRCA 1

MAP2K1

KEAP1

Bei einer Patientin mit einem pulmonalen Adenokarzinom mit *EGFR* p.L858R-Mutation tritt im Verlauf der Behandlung eine Resistenz gegenüber dem EGFR-Tyrosinkinaseinhibitor (TKI) auf. Sie werden gebeten, eine aktuelle Rebiopsie molekular zu untersuchen und detektieren eine Amplifikation des *MET*-Gens. Welche Information können Sie darauf basierend an die klinischen Kolleg*innen weitergeben?

Eine Fortführung der bisherigen EGFR-TKI-Therapie sollte mit einer angepassten Dosis erfolgen.

Die *MET*-Amplifikation stellt einen bekannten Resistenzmechanismus unter EGFR-TKI dar, bietet aber die Option einer alternativen/ergänzenden zielgerichteten Therapie.

Die *MET*-Amplifikation steht in keinem Zusammenhang mit der aufgetretenen Resistenz unter EGFR-TKI.

Die *MET*-Amplifikation ist prädiktiv für das Ansprechen gegenüber einer Immuncheckpointinhibitortherapie.

Es muss davon ausgegangen werden, dass der initial bestimmte *EGFR*-Mutationsstatus fehlerhaft ist, und eine erneute Analyse durchgeführt werden.

Welcher molekularpathologische Nachweis ist für die Indikationsstellung einer zugelassenen Therapie mit einem ROS1-Tyrosinkinaseinhibitor *am wenigsten* zielführend?

*ROS1*-Überexpression in der Immunhistochemie

*ROS1*-Fusion in der quantitativen Polymerasekettenreaktion mittels reverser Transkriptase (qRT-PCR)

Aktivierende *ROS1*-Punktmutation in der DNA-basierten Next-Generation-Sequencing(NGS)-Analyse

*ROS1*-Genumlagerung in der Fluoreszenz-in-situ-Hybridisierung

*ROS1*-Fusion in der RNA-basierten Next-Generation-Sequencing(NGS)-Analyse
