# Erratum zu: Molekularpathologie des nichtkleinzelligen Lungenkarzinoms: aktuelle und kommende Biomarker

**DOI:** 10.1007/s00292-025-01434-w

**Published:** 2025-04-23

**Authors:** Helen Pasternack, Jutta Kirfel

**Affiliations:** Institut für Pathologie, Universitätsklinikum Schleswig-Holstein, Campus Lübeck, Ratzeburger Allee 160, 23562 Lübeck, Deutschland

**Erratum zu:**

**Pathologie 2025**

10.1007/s00292-025-01433-x

Im Beitrag „Molekularpathologie des nichtkleinzelligen Lungenkarzinoms: aktuelle und kommende Biomarker“ wurden im Abschnitt „Alterationen von unmittelbarer klinischer Relevanz“ die Zwischenüberschriften *BRAF, MET, RET* und *ROS1* entfernt.

Der Originalbeitrag wurde korrigiert.

